# The impact of *Helicobacter pylori* eradication with vonoprazan-amoxicillin dual therapy combined with probiotics on oral microbiota: a randomized double-blind placebo-controlled trial

**DOI:** 10.3389/fmicb.2023.1273709

**Published:** 2023-10-02

**Authors:** Ruolin Peng, Zhenyu Zhang, Yi Qu, Weiwei Chen

**Affiliations:** Department of Gastroenterology, Nanjing First Hospital, Nanjing Medical University, Nanjing, China

**Keywords:** *Helicobacter pylori*, eradication, dual therapy, probiotics, oral microbiota

## Abstract

**Background:**

*Helicobacter pylori* infection and eradication have been reported to cause dysbiosis of the oral microbiota. Probiotics are increasingly being used to maintain the balance of the oral microbiota. We aimed to investigate the effects of *H. pylori* infection, *H. pylori* eradication with vonoprazan-amoxicillin dual therapy, and probiotics supplementation on the oral microbiota.

**Methods:**

*H. pylori* positive patients were randomly assigned to a vonoprazan-amoxicillin regimen plus probiotics (BtT group) or the placebo (PT group) for 14 days. *H. pylori* negative population served as normal controls. Tongue coating samples were collected from 60 *H. pylori* positive patients at three time points (before *H. pylori* eradication, after *H. pylori* eradication, and at confirmation of *H. pylori* infection cure) and 20 *H. pylori* negative subjects. 16S rRNA gene sequencing was used to analyze the oral microbiota.

**Results:**

*H. pylori* was detected in the oral cavity in positive (34/60), negative (7/20), and eradicated (1/60) subjects using high-throughput sequencing. Compared with normal controls, *H. pylori* positive patients exhibited higher richness (*p* = 0.012) and comparable diversity (*p* = 0.075) of oral microbiota. Beta diversity and KEGG analysis showed oral flora composition and function differences in *H. pylori* positive and negative subjects. Alpha diversity dramatically decreased after *H. pylori* eradication and modestly increased with confirmation of *H. pylori* eradication. Beta diversity and LEfSe analysis revealed distinct structures, and KEGG analysis showed distinct signaling pathways of tongue coating flora at three time points. There was a significant reduction of Firmicutes and *Lactobacillus* after *H. pylori* erdication. The PT group and BtT group had identical compositional and functional differences of oral microbiota at three time points.

**Conclusion:**

No substantial link existed between oral and stomach *H. pylori*, while removing gastric *H. pylori* helped eliminate oral *H. pylori*. *H. pylori* infection and vonoprazan-amoxicillin dual therapy affected oral microbiota diversity, structure, and function. *H. pylori* eradication demonstrated a suppressive impact on the proliferation of oral pathogens, specifically Firmicutes and *Lactobacillus*. Nevertheless, probiotics supplementation did not reduce the oral microbial disturbance caused by *H. pylori* eradication.

**Clinical trial registration:**

https://www.chictr.org.cn/, identifiers CHICTR2200060023.

## 1. Introduction

*Helicobacter pylori* is widely acknowledged as a pathogenic microorganism capable of causing damage to the stomach and various other physiological systems. The Kyoto Global Consensus (Sugano et al., 2015) recommends that all people infected with *H. pylori* should receive eradication therapy unless there are competing considerations. The oral cavity is situated proximal to the human gastrointestinal tract and serves as a potential reservoir of *H. pylori* (Al et al., 2014). Several studies have identified the presence of *H. pylori* in the oral cavity, specifically in dental plaque, saliva, and other samples (Ansari et al., 2018; Matamala-Valdes et al., 2018). Dysbiosis of the oral microbiota, a significant constituent of the human microbiome, has been observed to contribute to the development of various oral and systemic ailments, such as oral squamous cell carcinoma, diabetes, inflammatory bowel disease, and cardiovascular disease (Peng et al., 2022).

There have been reports indicating that *H. pylori* infection and subsequent eradication can alter the oral microbiota. The oral microbial community exhibited variations in diversity and composition among persons with *H. pylori* infection compared to those without infection (Chua et al., 2019; Hu et al., 2022), as well as before and after eradication occurred. *H. pylori* eradication with a 7-day triple therapy results in an instantaneous decline in the diversity of the oral flora (Jakobsson et al., 2010; Kadota et al., 2019). And this decline is accompanied by a drop in the abundance of oral pathogenic bacteria, as well as *Streptococcus* and *Actinomyces*. The restoration of oral flora diversity is observed to occur gradually following the completion of treatment. Ji et al. (2020) suggested that the implementation of quadruple bismuth therapy for *H. pylori* eradication led to a reduction in the oral flora’s diversity and an augmentation in the abundance of the Fusobacteriota, *Leptotrichia*, *Campylobacter*, and *Pseucomonas*. The study conducted by Hu et al. found that *H. pylori* eradication with vonoprazan-amoxicillin dual therapy for 7–10 days did not have a significant impact on the diversity of the oral microbiota. However, it was observed that this treatment led to changes in the composition of the flora, characterized by a reduction in the abundance of Firmicutes and Bacteroidota and an increase in the abundance of Proteobacteria (Hu et al., 2022).

Probiotics are described as “live microorganisms which when administered in adequate amounts confer a health benefit on the host” (Hill et al., 2014) and have been gradually proved to maintain the balance of oral microecology (Nguyen et al., 2021). Can probiotics supplementation mitigate oral microbiota disruption caused by *H. pylori* eradication therapy? To date, no relevant studies have been conducted to ascertain the answer. Therefore, we conducted a randomized, double-blind, placebo-controlled trial to explore the connection between *H. pylori* infection and eradication and oral microbiota. Additionally, we assessed the impact of probiotics supplementation on oral microbiology.

In our study, *H. pylori* positive patients were assigned randomly to receive a vonoprazan-amoxicillin regimen together with probiotics or the placebo for 14 days. Tongue coating samples were collected from *H. pylori* positive patients at three time points (before *H. pylori* eradication, after *H. pylori* eradication, and at confirmation of *H. pylori* infection cure) and 20 *H. pylori* negative subjects. 16S rRNA gene sequencing was used to evaluate the structure and function of the samples.

## 2. Materials and methods

### 2.1. Study design

The study was designed as a single-center, randomized, double-blind, placebo-controlled, and prospective clinical trial, adhering to the principles outlined in the Declaration of Helsinki, which obtained approval from the Medical Ethics Committee of the Nanjing First Hospital (KY20220314-05) and registered with the Chinese clinical trial registry (CHICTR2200060023).

From December 27, 2021 to June 22, 2022, a cohort of 100 *H. pylori* positive patients, ranging in age from 18 to 65, diagnosed by 13C-UBT (DOB > 6‰), histology, or culture, were recruited from Nanjing First Hospital in China. The main exclusion criteria were as follows: (1) allergy to the study drug; (2) patients with chronic gastritis with a confirmed peptic ulcer; (3) patients who had previously received *H. pylori* eradication therapy within 6 months; (4) use of antibiotics, bismuth, and probiotics within 4 weeks or H2 receptor antagonists, proton pump inhibitor (PPI), and potassium competitive acid blockers (P-CAB) within 2 weeks; (5) use of adrenocortical hormones, non-steroidal anti-inflammatory drugs, or anticoagulants; (6) history of esophageal or gastric surgery; (7) pregnant or lactating women; (8) suffer from a serious illness such as liver disease, cardiovascular disease, lung disease, kidney disease, or tumor; (9) alcoholism. Twenty *H. pylori* negative healthy volunteers (DOB < 4‰ of 13C-UBT) were selected as normal controls who had no history of smoking or alcohol consumption and oral disease.

### 2.2. Randomization and double-blinding

The biostatisticians programmed the generation of the random allocation sequence for this clinical trial using the SAS 9.4 statistical analysis system and achieved it through the block randomization method with a block length of 4. Subjects meeting the enrollment criteria were randomly assigned to receive the vonoprazan-amoxicillin regimen along with probiotics in the BtT group or the placebo in the PT group for 14 days in a 1:1 ratio. The study medicine was administered to enrolled subjects based on their given randomization number, ensuring that both the subjects and the administering researchers remained blinded.

### 2.3. Intervention and control

The BtT group was administered a regimen consisting of amoxicillin 750 mg qid, vonoprazan 20 mg bid, and Bifidobacterium tetravaccine tablets (3 tablets tid) for 14 days. The probiotics contained 0.5 g per tablet. The PT group received the identical vonoprazan-amoxicillin dual therapy in conjunction with the placebo, which was made of starch and had the same weight, appearance, and taste as Bifidobacterium tetravaccine tablets, except that it lacked the active ingredient. Both the probiotics and placebo were provided by Hangzhou Grand Biologic-Pharmaceutical, Inc. And the probiotics or placebo must be taken more than 2 h apart from amoxicillin.

### 2.4. Sample collection

Tongue coating samples were collected from *H. pylori* positive patients at three time points (before *H. pylori* eradication (W0), after *H. pylori* eradication (W2), and at confirmation of *H. pylori* infection cure (W6)) and *H. pylori* negative patients. Before the collection process, the oral cavity of each individual was washed with a normal saline solution two to three times. Sterile cotton swabs were utilized to collect samples from a specific region measuring 2 cm × 2 cm located in the central section of the dorsal surface of the tongue. Following 30 s of wiping, the samples were subsequently transferred into sterile test tubes and stored in a refrigerator set at a temperature of −80°C until they were deemed suitable for use.

### 2.5. DNA extraction and sequencing

Total genomic DNA was isolated using the CTAB technique. The concentration and purity of the DNA were evaluated on a 1% agarose gel. Specific primers (341F, CCTAYGGGRBGCASCAG, 806R, GGACTACNGGGTATTAAT) and barcodes were applied to amplify 16S genes from separate locations (V3-V4). All PCR reactions were performed using 15 μL of Phusion® High-Fidelity PCR Master Mix (New England Biolabs), 0.2 μM of forward and reverse primers, and roughly 10 ng of template DNA. The thermal cycle underwent initial denaturation at 98°C for 1 min, followed by denaturation at 98°C for 10 s, annealing at 50°C for 30 s, elongation at 72°C for 30 s, and finally elongation at 72°C for 5 min. The same amount of IX loading buffer (containing SYB green) was mixed with PCR products and detected by electrophoresis on a 2% agarose gel. The PCR products were mixed at an equal density ratio, then purified with the Qiagen Gel Extraction Kit (Qiagen, Germany). Sequencing libraries were generated using the TruSeq® DNA PCR-Free Sample Preparation Kit (Illumina, United States) following the manufacturer’s recommendations, and index codes were added. Finally, 250 bp paired-end reads were acquired by sequencing on the Illumina NovaSeq platform.

### 2.6. Sequencing analysis

First, the technician screened the raw data and discarded any sequences that were shorter than 230 bp, had a quality score of ≤20, contained ambiguous bases, and did not precisely match the primer and barcode tag sequences, and then separated the sequences using sample-specific barcode tags. Then, the high-quality sequences were de-noised using the unoise3 method with usearch11 to produce amplicon sequence variants (ASVs; Edgar, 2016). Finally, all sequences were classified into different taxonomic groups against the SILVA138 database by the BLAST tool.

### 2.7. Bioinformatics and statistical analysis

Qiime (v1.8.0) was used to calculate the richness and diversity indices (Chao1 richness index and Shannon diversity index) based on the ASV information. To compare the membership and structure of microbial communities in different samples, heat maps of the top 20 ASVs were generated using Mothur (Jami et al., 2013). According to the results of taxonomic annotation and relative abundance, PCoA based on bray-curtis distance and PLS-DA were performed using R packages (v3.6.0) to test the similarity between different samples (Wang et al., 2012). LEfSe was used to measure the unique species between *H. pylori* positive and negative groups, as well as before and after the *H. pylori* eradication. LDA value >3.0 was considered statistically significant. Microbial function was predicted by using PICRUST2 in the KEGG database.^1^ Co-occurrence network analysis was performed by the spearman method.

SPSS 25.0 statistical software was used to process the data, and the clinical data were compared by analysis of variance. Continuous data were presented as mean ± standard deviation. Disaggregated information is expressed as frequencies or percentages. Comparisons of measured information among groups were analyzed by one-way ANOVA, and categorical information was analyzed by Chi-squared test. When *p* < 0.05, the data exhibited statistical significance.

## 3. Results

### 3.1. Features of subjects

**Figure 1:**
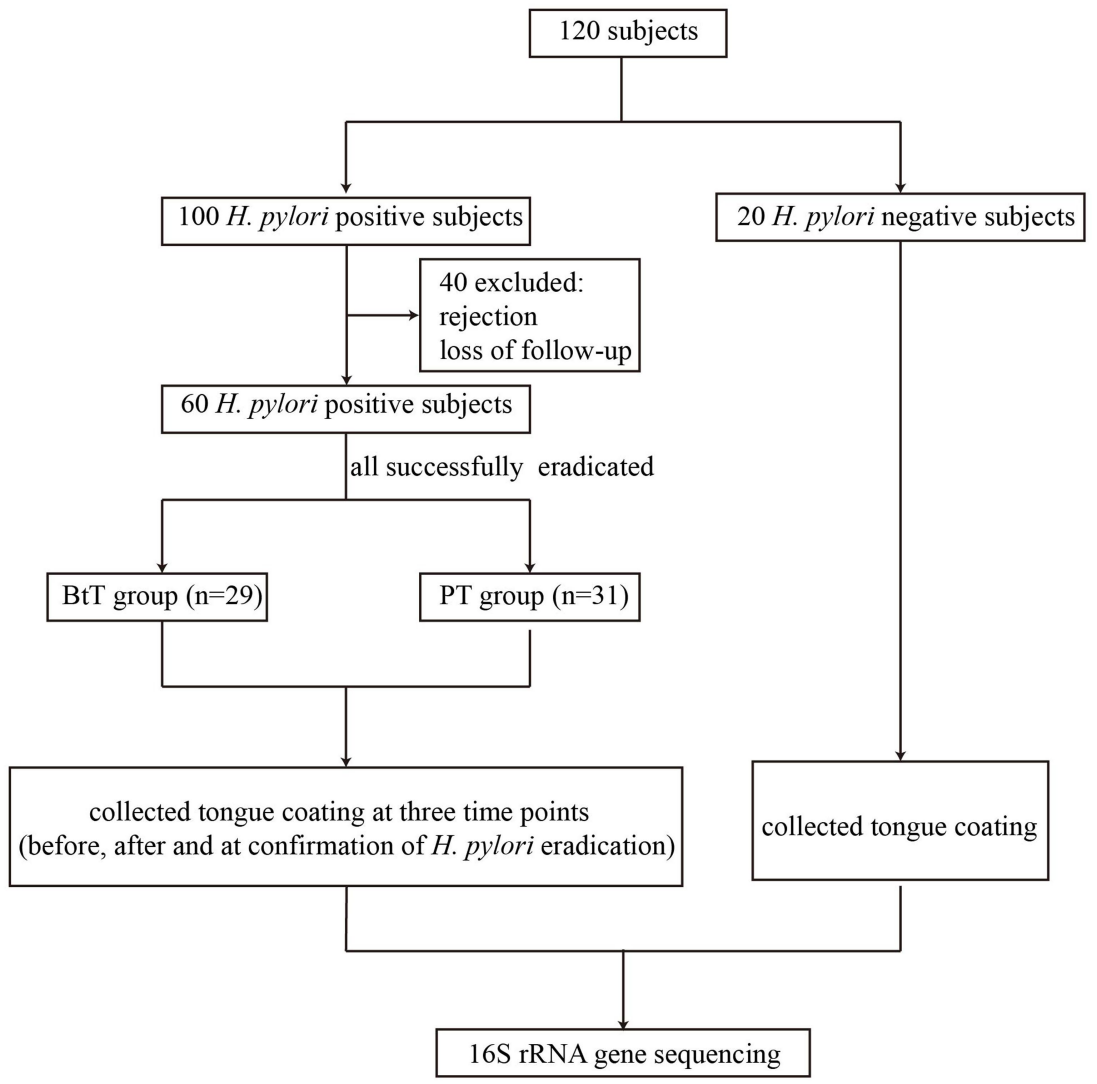
Flow chart of the study. BtT group: vonoprazan-amoxicillin regimen plus probiotics. PT group: vonoprazan-amoxicillin regimen plus the placebo.

A total of 120 participants were included in the study, consisting of 100 *H. pylori* positive patients and 20 *H. pylori* negative individuals. Tongue coating samples were not collected from 40 *H. pylori* positive patients because of rejection or loss of follow-up. Finally, 60 *H. pylori* positive patients (referred to as the Hp group) who had undergone successful *H. pylori* eradication treatment, with 29 patients belonging to the BtT group and 31 patients belonging to the PT group, and 20 *H. pylori* negative individuals (referred to as the NT group) were selected for oral microbiota analysis (Figure 1).

There were no significant differences among the BtT group, PT group, and NT group in demographic characteristics such as age, sex, BMI index, smoking, and alcohol (Supplementary Table S1).

### 3.2. Detection of oral *Helicobacter pylori*

*H. pylori* was detected in the oral cavity of infected subjects (34/60, 56.7%) and uninfected subjects (7/20, 35%) with no statistical differences (*p* > 0.05). The detection rate among eradicated subjects was 1.7% (1/60) by high-throught sequencing, significantly lower than the other two groups (*p* < 0.01).

### 3.3. *Helicobacter pylori* infection and oral microbiota

#### 3.3.1. Diversity analysis

**Figure 2:**
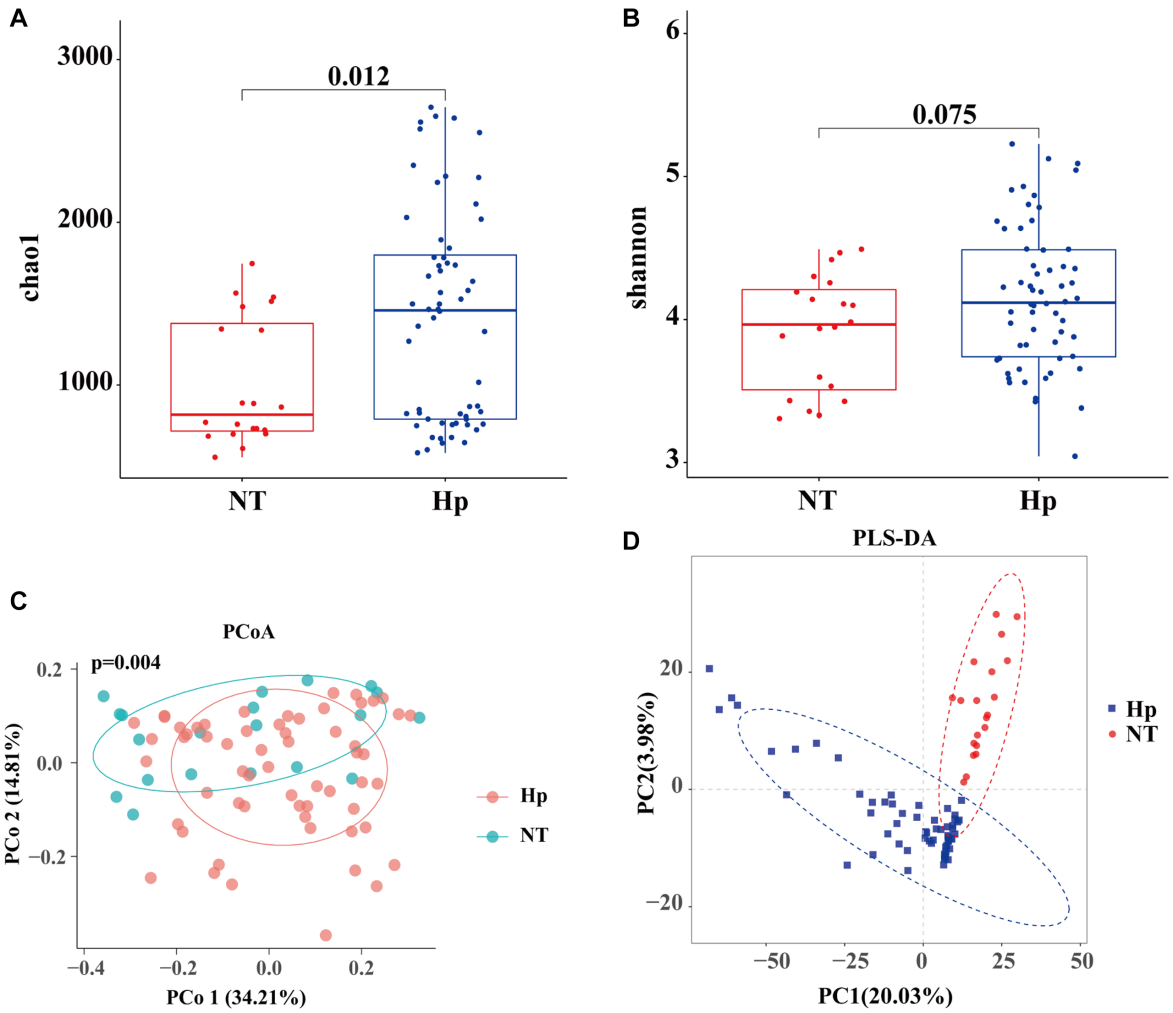
Diversity of oral microbiota between *H. pylori* positive and negative subjects. The Hp group exhibited a higher richness compared to the NT group **(A)**, while there was no significant difference in diversity **(B)**. The PCoA analysis using the bray-curtis distance metric **(C)** and the PLS-DA results **(D)** revealed significant dissimilarity in community structure between the two groups. Hp: *H. pylori* positive. NT: *H. pylori* negative.

The Chao1 richness index (Figure 2A) and Shannon diversity index (Figure 2B) were used to assess the alpha diversity of the tongue coating flora. The results indicated that the Hp group exhibited a higher richness compared to the NT group (Chao1 index: 1418.24 vs. 1006.72, *p* = 0.012). However, there was no statistically significant difference in diversity between the two groups (Shannon index: 4.15 vs. 3.91, *p* = 0.075). The PCoA analysis, using the bray-curtis distance metric (Figure 2C), revealed significant dissimilarity in community structure between the two groups (*p* = 0.001). In addition, the results obtained from PLS-DA revealed clear disparities in the taxonomic composition between the two groups, as depicted in Figure 2D.

#### 3.3.2. Bacterial abundance and composition analysis

**Figure 3:**
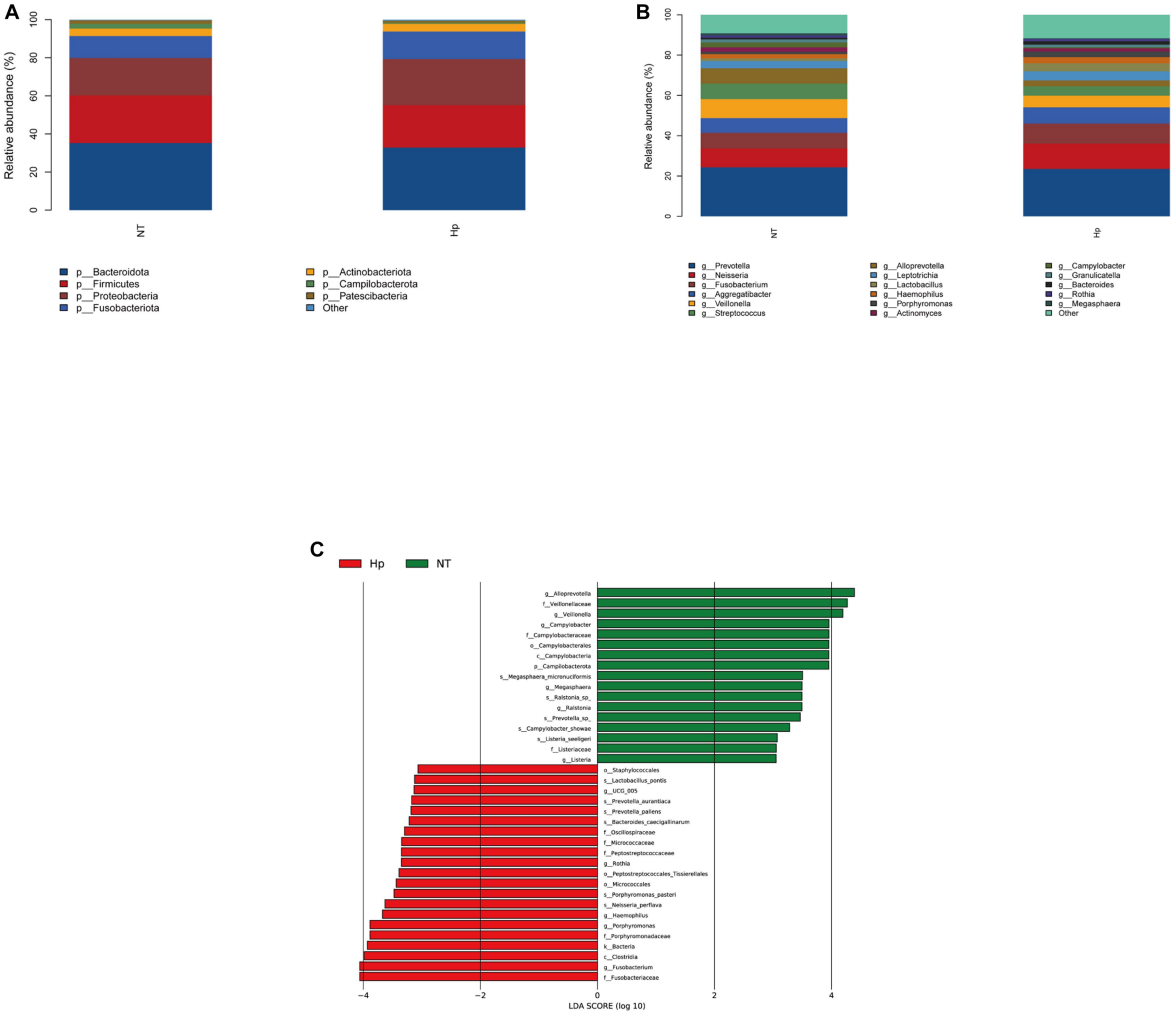
Composition of oral microbiota between *H. pylori* positive and negative subjects. The structure of oral microbiota between the two groups at the phylum level **(A)** and the genus level **(B)**. Different specific oral bacteria assessed by LEfSe between the two groups **(C)**. Hp: *H. pylori* positive. NT: *H. pylori* negative.

At the phylum level, the five dominant bacteria in the Hp group and the NT group both were Bacteroidota, Firmicutes, Proteobacteria, Fusobacteriota, and Actinobacteriota (Figure 3A), together accounting for more than 95% of the total sequences. Table 1 displayed the relative abundance of the predominant phyla (relative abundance ≥1% of the total sequences) in both groups. In comparison to the NT group, the Hp group exhibited a decrease in the relative abundance of Bacteroidota, Firmicutes, and Patescibacteria (35.2% vs. 32.9, 25.0% vs. 22.2%, and 1.8% vs. 1.1%, respectively). Conversely, the Hp group showed an increase in the relative abundance of Proteobacteria, Fusobacteriota, and Actinobacteriota (19.7% vs. 24.2%, 11.5% vs. 14.4%, and 3.9% vs. 4.0%, respectively). However, these differences were not statistically significant (*p* > 0.05). The only significant difference was observed in Campilobacterota, where the relative abundance was much lower in the Hp group compared to the NT group (0.6% vs. 2.5%, *p* = 0.00).

**Table 1:** Comparison of the relative abundance of dominant phyla (relative abundance ≥1% of the total sequences) between *H. pylori* positive and negative subjects.

Dominant phyla	Hp group	NT group	*p* value
Bacteroidota	0.32854	0.35233	0.34494
Firmicutes	0.22233	0.24972	0.25707
Proteobacteria	0.24235	0.19714	0.10475
Fusobacteriota	0.14441	0.11472	0.06842
Actinobacteriota	0.04026	0.03945	0.31196
Campilobacterota	0.00558^a^	0.02497	0.00000^*^
Patescibacteria	0.01066	0.01817	0.76417

The relative abundance of Campilobacterota was significantly lower in the Hp group than in the NT group. ^*^p < 0.01. ^a^Relative abundance <1% of the total sequences in this group. Hp group: *H. pylori* positive subjects. NT group: *H. pylori* negative subjects.

As illustrated in Figure 3B, the top ten dominant genera within the NT group were identified as *Prevotella*, *Neisseria*, *Veillonella*, *Fusobacterium*, *Streptococcus*, *Alloprevotella*, *Aggregatibacter*, *Leptotrichia*, *Campylobacter*, and *Actinomyces*, accounting for 58.6% (top five dominant genera) and 81.7% (top ten dominant genera) of the total sequences. The top ten dominant genera within the Hp group were ranked as follows: *Prevotella*, *Neisseria*, *Fusobacterium*, *Aggregatibacter*, *Veillonella*, *Streptococcus*, *Leptotrichia*, *Lactobacillus*, *Haemophilus*, and *Alloprevotella*, accounting for 59.9% (top five dominant genera) and 79.0% (top ten dominant genera) of the total sequences. The comparison of the relative abundance of the top ten dominant genera between the two groups, as shown in Table 2, indicated that there were significant differences in the relative abundance of certain bacteria. Specifically, the relative abundance of *Veillonella*, *Alloprevotella*, and *Campylobacter* was significantly lower in the Hp group compared to the NT group (5.8% vs. 9.4, 2.9% vs. 7.6%, and 0.5% vs. 2.5%, respectively, *p* < 0.05). On the other hand, the relative abundance of *Fusobacterium* and *Haemophilus* was significantly higher in the Hp group compared to the NT group (9.9% vs. 7.8% and 3.0% vs. 2.0%, respectively, *p* < 0.05).

**Table 2:** Comparison of the relative abundance of the top ten dominant genera between *H. pylori* positive and negative subjects.

Dominant genera	Hp group	NT group	*p* value
*Prevotella*	0.23540	0.24287	0.85890
*Neisseria*	0.12580	0.09376	0.07186
*Veillonella*	0.05827	0.09369	0.04922^*^
*Fusobacterium*	0.09894	0.07810	0.02781^*^
*Streptococcus*	0.04601	0.07748	0.21952
*Alloprevotella*	0.02906	0.07605	0.00260^*^
*Aggregatibacter*	0.08047^a^	0.07304	0.16151
*Leptotrichia*	0.04539	0.03628	0.07012
*Campylobacter*	0.00537^a^	0.02491	0.00000^*^
*Actinomyces*	0.01556	0.02112	0.92917
*Lactobacillus*	0.04013	0.01339^a^	0.20287
*Haemophilus*	0.03005	0.02003^a^	0.00216^*^

The relative abundance of *Veillonella*, *Alloprevotella*, and *Campylobacter* was significantly lower, whereas the relative abundance of *Fusobacterium* and *Haemophilus* was significantly higher in the Hp group compared with the NT group. ^*^p < 0.05. ^a^Not belong to one of the top ten dominant genera in this group. Hp group: *H. pylori* positive subjects. NT group: *H. pylori* negative subjects.

The LEfSe method was employed to identify distinct oral bacterial taxa present in both the Hp group and the NT group (Figure 3C). The potential biomarkers identified in this study encompassed *Ruminococcaceae UCG-005*, *Rothia*, *Haemophilus*, *Porphyromonas*, *Fusobacterium*, and *Haemophilus* of the Hp group, as well as Campilobacterota, *Alloprevotella*, *Veillonella*, *Campylobacter*, *Megasphaera*, *Ralstonia*, and *Listeria* of the NT group.

#### 3.3.3. Functional analysis

The results of the KEGG analysis (Supplementary Figure S1) demonstrated notable disparities in signaling pathways between the Hp group and the NT group. *H. pylori* positive subjects showed enriched signaling pathways associated with cardiovascular disease, bacterial infectious disease, and viral infectious disease. Conversely, the signaling pathway related to lipid metabolism showed a decrease in activity.

### 3.4. *Helicobacter pylori* eradication with vonoprazan-amoxicillin dual therapy and oral microbiota

#### 3.4.1. Diversity analysis

**Figure 4:**
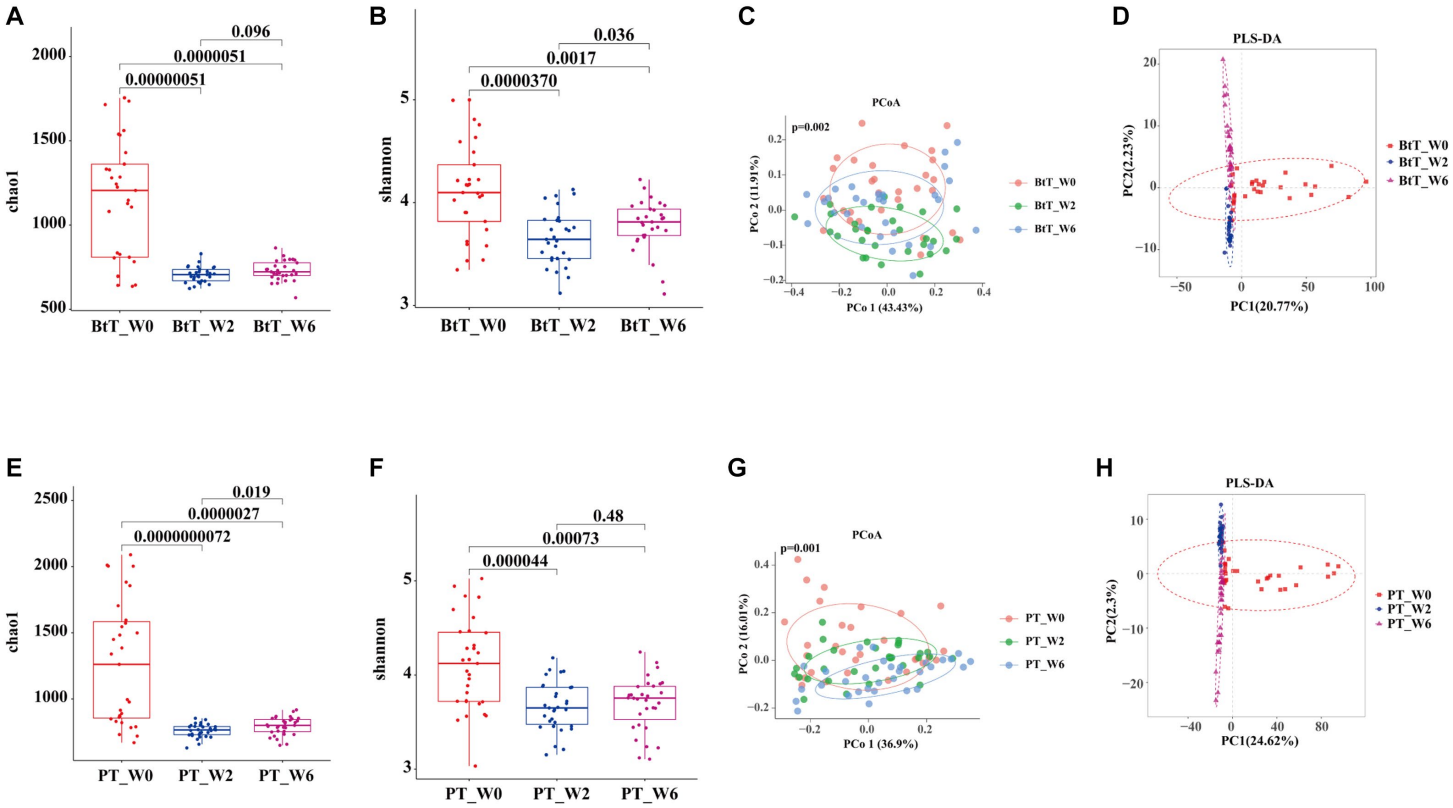
Diversity of oral microbiota at W0, W2, and W6 of the BtT group and the PT group. In the BtT group, the richness **(A)** and diversity **(B)** of tongue coating flora decreased significantly at W2 and slightly increased at W6. The PCoA analysis based on bray-curtis distance **(C)** and the PLS-DA results **(D)** revealed a clear difference in oral microbial structure in the BtT group at W0, W2, and W6. In the PT group, the richness **(E)** and diversity **(F)** of tongue coating flora also declined significantly at W0 and somewhat rose at W6. There was a distinct difference in oral microbial structure in the PT group at W0, W2, and W6 by the beta analysis of PCoA based on bray-curtis distance **(G)** and the PLS-DA results **(H)**. BtT group: vonoprazan-amoxicillin regimen plus probiotics. PT group: vonoprazan-amoxicillin regimen plus the placebo. W0: before *H. pylori* eradication. W2: after *H. pylori* eradication. W6: at confirmation of *H. pylori* infection cure.

We successfully acquired 180 tongue coating samples from 29 subjects in the BtT group and 31 subjects in the PT group at three different time points: W0, W2, and W6. The Chao1 and Shannon indices were utilized to depict the richness and diversity of the two groups, as illustrated in Figures 4A,B,E,F, respectively. In the BtT group, there was a notable drop in the alpha diversity of tongue coating flora at W2 (Chao1 index: 1150.30 vs. 706.00, *p* = 0.00000051; Shannon index: 4.12 vs. 3.66, *p* = 0.000037). However, there was a little rise in alpha diversity at W6 (Chao1 index: 706.00 vs. 728.43, *p* = 0.096; Shannon index: 3.66 vs. 3.78, *p* = 0.036). The alpha diversity of tongue coating flora in the PT group exhibited a substantial decrease at W0 (Chao1 index: 1265.99 vs. 759.87, *p* = 0.0000000072; Shannon index: 4.11 vs. 3.66, *p* = 0.000044) and a slight increase at W6 (Chao1 index: 759.87 vs. 794.55, *p* = 0.019; Shannon index: 3.66 vs. 3.69, *p* = 0.48).

The beta analysis of PCoA, based on bray-curtis distance, demonstrated a distinct dissimilarity in the composition of oral microbiota between the two groups at W0, W2, and W6 (Figures 4C,G). According to the PLS-DA results, it was observed that the two groups exhibited distinctive clustering at W0, W2, and W6 (Figures 4D,H).

#### 3.4.2. Bacterial abundance and composition analysis

**Figure 5:**
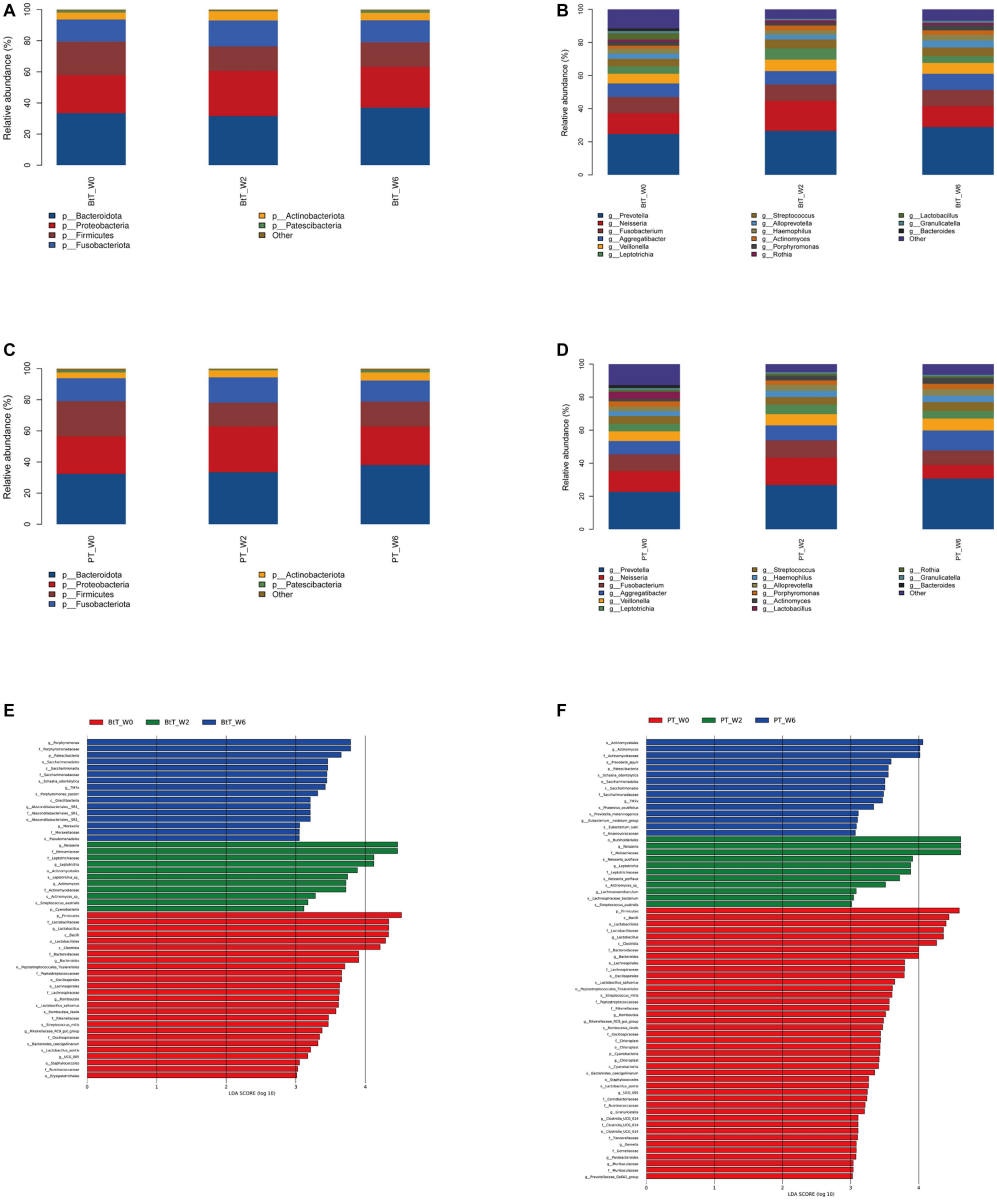
Composition of oral microbiota at W0, W2, and W6 of the BtT group and the PT group. Composition at the phylum level **(A)** and at the genus level **(B)** of the BtT group. Composition at the phylum level **(C)** and at the genus level **(D)** of the PT group. The distinct oral microorganisms of the BtT group **(E)** and the PT group **(F)** assessed by LEfSe analysis. BtT group: vonoprazan-amoxicillin regimen plus probiotics. PT group: vonoprazan-amoxicillin regimen plus the placebo. W0: before *H. pylori* eradication. W2: after *H. pylori* eradication. W6: at confirmation of *H. pylori* infection cure.

At the phylum level, the top five dominant bacteria of the BtT group (Figure 5A) and the PT group (Figure 5C) at W0, W2, and W6 all were Bacteroidota, Proteobacteria, Firmicutes, Fusobacteriota, and Actinobacteriota, together accounting for more than 97% of the total sequences. The relative abundance of the top five prevalent phyla at W0, W2, and W6 was compared and presented in Table 3 (BtT group) and Table 4 (PT group), which suggested a considerable drop in the relative abundance of Firmicutes at W2, followed by a slight increase at W6. There were no statistically significant differences in the relative abundance of the other four main phyla.

**Table 3:** Comparison of the relative abundance of the top five dominant phyla at W0, W2, and W6 of the BtT group.

Dominant phyla	BtT-W0	BtT-W2	BtT-W6	*p* value
Bacteroidota	0.33459	0.31587	0.36970	0.24681
Proteobacteria	0.24416	0.29049	0.26257	0.39145
Firmicutes	0.21556	0.15763	0.15855	0.00270^*^
Fusobacteriota	0.14238	0.16680	0.14047	0.32283
Actinobacteriota	0.04412	0.05838	0.04801	0.12408

The relative abundance of Firmicutes decreased significantly at W2 and increased littlely at W6. ^*^p < 0.01. BtT group: vonoprazan-amoxicillin regimen plus probiotics. W0: before *H. pylori* eradication. W2: after *H. pylori* eradication. W6: at confirmation of *H. pylori* infection cure.

**Table 4:** Comparison of the relative abundance of the five dominant phyla at W0, W2, and W6 of the PT group.

Dominant phyla	PT-W0	PT-W2	PT-W6	*p* value
Bacteroidota	0.32343	0.33412	0.38089	0.21640
Proteobacteria	0.24132	0.29485	0.24667	0.16083
Firmicutes	0.22663	0.15212	0.16107	0.00031^*^
Fusobacteriota	0.14710	0.16261	0.13511	0.17302
Actinobacteriota	0.03695	0.04536	0.05207	0.19966

The relative abundance of Firmicutes decreased significantly at W2 and increased littlely at W6. ^*^p < 0.01. PT group: vonoprazan-amoxicillin regimen plus the placebo. W0: before *H. pylori* eradication. W2: after *H. pylori* eradication. W6: at confirmation of *H. pylori* infection cure.

At the genus level, *Prevotella*, *Neisseria*, *Fusobacterium*, *Aggregatibacter*, and *Veillonella* were identified as the five dominant bacteria in both the BtT group (Figure 5B) and the PT group (Figure 5D) at W0, W2, and W6. The relative abundance of the top ten genera at W0, W2, and W6 was compared and presented in Table 5 (BtT group) and Table 6 (PT group). The results indicated that around 50% of the dominant genera exhibited similar levels of abundance throughout all three time points. Nevertheless, there were notable distinctions observed among *Leptotrichia*, *Actinomyces*, *Lactobacillus*, *Porphyromonas*, and *Neisseria*. Within the BtT group, there was a marginal rise in the relative abundance of *Leptotrichia*, *Neisseria*, and *Actinomyces*, which subsequently reverted back to the baseline level at W6. Following the therapy, there was a persistent drop in the relative abundance of *Lactobacillus*. Additionally, the relative abundance of *Porphyromonas* exhibited a modest decline at W2 but subsequently surpassed the baseline level at W6. In the PT group, the relative abundance of *Leptotrichia* slightly rose at W2 and decreased to the baseline level at W6. In addition, the abundance of *Actinomyces* exhibited a progressive increase following the therapy. In contrast, the relative abundance of *Lactobacillus* exhibited a notable decrease subsequent to the treatment, while the abundance of *Neisseria* experienced a minor increase at W2, followed by a subsequent decline below the starting level at W6.

**Table 5:** Comparison of the relative abundance of the ten dominant genera at W0, W2, and W6 of the PT group.

Dominant genera	BtT-W0	BtT-W2	BtT-W6	*p* value
*Prevotella*	0.24653	0.26584	0.28891	0.57115
*Neisseria*	0.12623	0.18074	0.12515	0.04156^*^
*Fusobacterium*	0.09732	0.09904	0.09887	0.96022
*Aggregatibacter*	0.08258	0.08131	0.09733	0.56033
*Veillonella*	0.05829	0.06935	0.06639	0.06847
*Streptococcus*	0.04551	0.05452	0.05164	0.63918
*Leptotrichia*	0.04498	0.06768	0.04155	0.00156^*^
*Lactobacillus*	0.03844	0.00002^a^	0.00002^a^	0.00000^*^
*Alloprevotella*	0.03099	0.02793	0.04165	0.27320
*Haemophilus*	0.02989	0.02610	0.03460	0.42367
*Porphyromonas*	0.02448^a^	0.01819^a^	0.03230	0.03445^*^
*Actinomyces*	0.01812^a^	0.02966	0.02732^a^	0.01230^*^

The relative abundance of *Leptotrichia*, *Neisseria,* and *Actinomyces* showed a slight increase at W2, but returned to the baseline level by W6. The relative abundance of *Lactobacillus* continued to decrease after treatment, and the relative abundance of *Porphyromonas* declined slightly at W2 but exceeded the baseline level at W6. ^*^p < 0.05. ^a^Not belong to one of the top ten predominant genera at this time point. BtT group: vonoprazan-amoxicillin regimen plus probiotics. W0: before *H. pylori* eradication; W2: after *H. pylori* eradication; W6: at confirmation of *H. pylori* infection cure.

**Table 6:** Comparison of the relative abundance of the ten dominant genera at W0, W2, and W6 of the PT group.

Dominant genera	PT-W0	PT-W2	PT-W6	*p* value
*Prevotella*	0.22629	0.26801	0.30685	0.06551
*Neisseria*	0.12607	0.16502	0.08233	0.00014^*^
*Fusobacterium*	0.10133	0.10563	0.08813	0.43040
*Aggregatibacter*	0.08052	0.09039	0.12080	0.20313
*Veillonella*	0.05873	0.06828	0.07242	0.28534
*Streptococcus*	0.04683	0.04647	0.05179	0.60997
*Leptotrichia*	0.04567	0.05690	0.04689	0.01278^*^
*Lactobacillus*	0.04190	0.00002^a^	0.00004^a^	0.00000^*^
*Porphyromonas*	0.03278	0.02687^a^	0.03369^a^	0.45412
*Haemophilus*	0.02901	0.03761	0.04044	0.30151
*Alloprevotella*	0.02735^a^	0.03636	0.03771	0.14689
*Actinomyces*	0.01540^a^	0.02804	0.03584	0.00254^*^

The relative abundance of *Leptotrichia* slightly rose at W2 and decreased to the baseline level at W6, and *Actinomyces* abundance gradually increased after treatment. Otherwise, the relative abundance of *Lactobacillus* dropped significantly after treatment, while *Neisseria* abundance slightly increased at W2 and then fell below the initial level at W6. ^*^p < 0.05. ^a^Not belong to one of the top ten dominant genera at this time point. PT group: vonoprazan-amoxicillin regimen plus the placebo. W0: before *H. pylori* eradication; W2: after *H. pylori* eradication; W6: at confirmation of *H. pylori* infection cure.

We further performed co-occurrence network analysis to assess the impact of eradication treatment on oral microbiota interactions at W0 and W2 in the BtT group (Supplementary Figure S2) and in the PT group (Supplementary Figure S3). A negative association was seen between the abundance of *Lactobacillus* and the presence of *Leptotrichia*, *Neisseria*, and *Actinomyces* in both groups. This finding provided additional support for the previously identified variance in bacterial abundance.

The distinct oral microorganisms screened by LEfSe analysis at W0, W2, and W6 were shown in Figure 5E (BtT group) and Figure 5F (PT group). The potential biomarkers identified in the BtT group encompassed many taxonomic groups, including Firmicutes, *Lactobacillus*, *Bacteroides*, *Romboutsia*, and *Rikenellaceae RC9* (at W0). At W2, the biomarkers consisted of Cyanobacteria, *Neisseria*, *Leptotrichia*, and *Actinomyces*. Finally, at W6, the biomarkers included Patescibacteria, *Porphyromonas*, *TM7x*, *Absconditabacteriales SR1*, and *Moraxella*. The potential biomarkers identified in the PT group were as follows: Firmicutes, Cyanobacteria, *Lactobacillus*, *Bacteroides*, *Romboutsia*, *Rikenellaceae_RC9_gut_group*, Chloroplast, *Granulicatella*, *Ruminococcaceae UCG-005*, *Clostridia_UCG − 014*, *Parabacteroides*, *Prevotellaceae_Ga6A1_group*, *Muribaculaceae*, *Gemella* (at W0), and *Neisseria*, *Leptotrichia*, *Lachnoanaerobaculum* (at W2), and Patescibacteria, *Actinomyces*, *Eubacterium_nodatum_group*, *TM7x* (at W6).

### 3.5. Functional analysis

**Figure 6:**
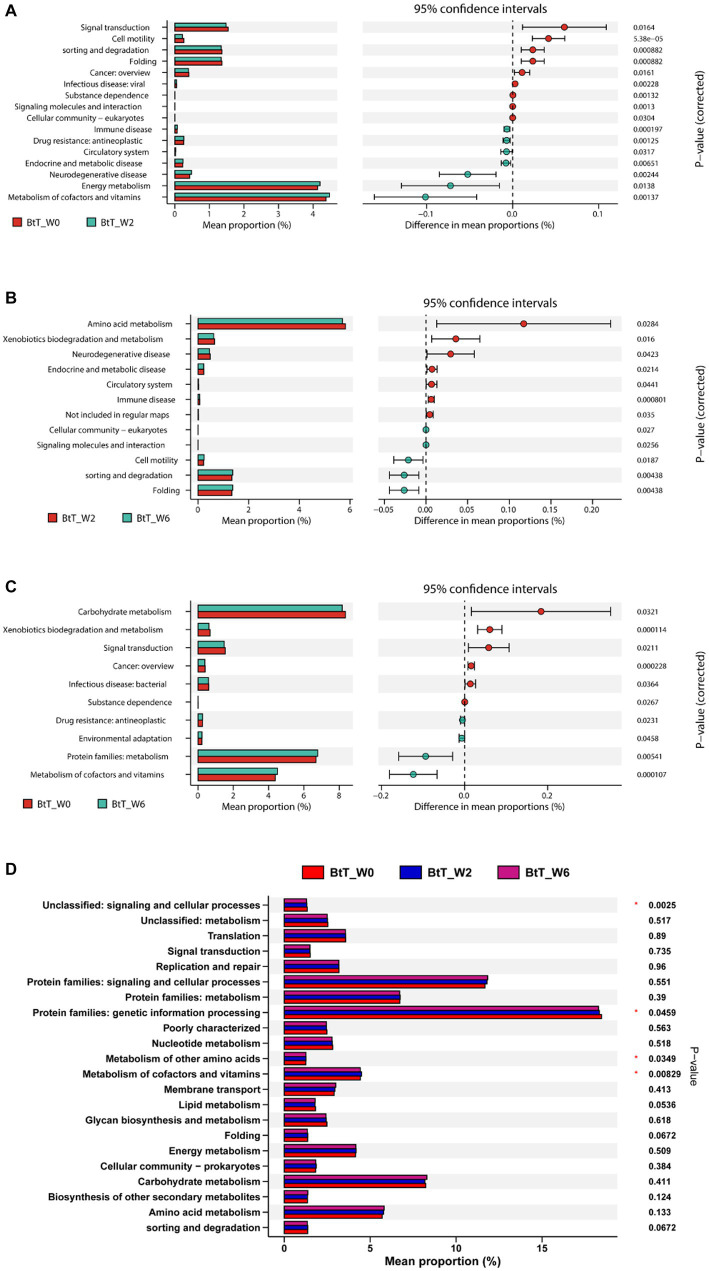
The KEGG analysis before and after *H. pylori* eradication of the BtT group. There were significant variations in the signaling pathways of 16, 12, and 10, respectively, between the time points W0 and W2 **(A)**, W2 and W6 **(B)**, and W0 and W6 **(C)** in the BtT group. And there was a significant difference between 4 pathways in the BtT group at W0, W2, and W6 **(D)**. BtT group: vonoprazan-amoxicillin regimen plus probiotics. W0: before *H. pylori* eradication. W2: after *H. pylori* eradication. W6: at confirmation of *H. pylori* infection cure.

**Figure 7:**
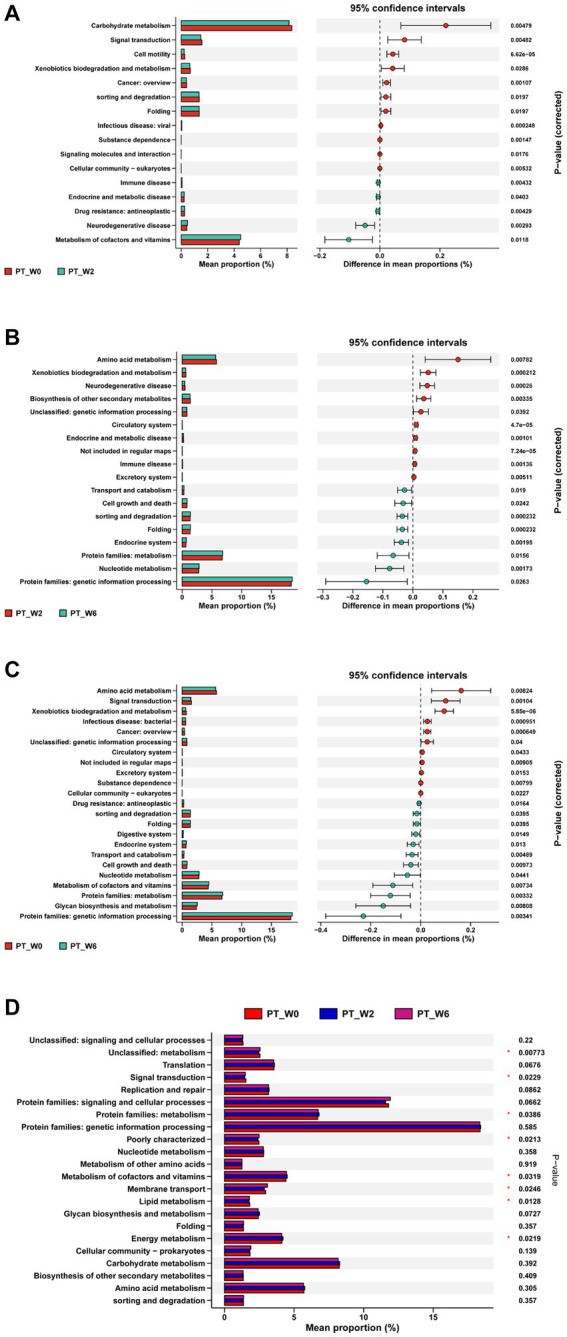
The KEGG analysis before and after *H. pylori* eradication of the PT group. The PT group exhibited variations of 16, 18, and 23 routes at W0 and W2 **(A)**, W2 and W6 **(B)**, and W0 and W6 **(C)**, respectively. And there was a significant difference between 8 pathways in the PT group at W0, W2, and W6 **(D)**. PT group: vonoprazan-amoxicillin regimen plus the placebo. W0: before *H. pylori* eradication. W2: after *H. pylori* eradication. W6: at confirmation of *H. pylori* infection cure.

The KEGG analysis demonstrated statistically significant variations in the signaling pathways of 16, 12, and 10, respectively, between the time points W0 and W2 (Figure 6A), W2 and W6 (Figure 6B), and W0 and W6 (Figure 6C) in the BtT group. The PT group exhibited variations of 16, 18, and 23 routes at W0 and W2 (Figure 7A), W2 and W6 (Figure 7B), and W0 and W6 (Figure 7C), respectively. The dissimilarities in signaling pathways between W0 and W2 primarily included genetic information processing, environmental information processing, celluar process, human diseases, and metabolism. After *H. pylori* eradication, there was a decrease in the signaling pathways associated with genetic information processing (including folding and sorting and degradation), environmental information processing (including signal transduction and signaling molecules and interaction), cellular process (including cellular motility and cellular community), and human diseases (including cancer, viral infectious disease, and substance dependence). Conversely, the signaling pathways involved in metabolism (including metabolism of cofactors and vitamins) and human diseases (including drug resistance: antineoplastic, endocrine and metabolic disease, immune disease, and neurodegenerative disease) experienced a decrease. The aforementioned modifications were partially reverted to their original levels. Moreover, notable disparities were seen between 4 signaling pathways in the BtT group (Figure 6D) and 8 signaling pathways in the PT group (Figure 7D) at W0, W2, and W6.

## 4. Discussion

To our best knowledge, this is the first clinical trial to explore the influence of vonoprazan-amoxicillin dual therapy combined with probiotics for 14 days on the alteration of the oral microbiota. Simultaneously, we investigated the relationship between the existence of oral *H. pylori* and the status of *H. pylori* infection in the stomach, as well as the impact of *H. pylori* infection on the oral microbiota. The novel discoveries were articulated in the following manner.

### 4.1. The relationship between oral *Helicobacter pylori* and gastric *Helicobacter pylori*

*H. pylori* is a gram-negative bacteria that predominantly inhabits in the epithelial and mucus layers of the gastric mucosa and can be transmitted by both the oral-oral and fecal-oral routes, which establishes the oral cavity as a possible reservoir of *H. pylori* (Al et al., 2014). Previous studies have identified the presence of *H. pylori* in dental plaque (Mendoza-Cantu et al., 2017), saliva (Seligova et al., 2020), tongue coating (Zhao et al., 2019), and dental pulp (Nomura et al., 2018). Nevertheless, the association between oral *H. pylori* and gastric *H. pylori* is still not well understood. According to a study (Yee, 2016), there was a notable disparity in the detection rate of oral *H. pylori* between *H. pylori* positive patients and *H. pylori* negative people (80% vs. 23%), suggesting a potential association between gastric *H. pylori* and the occurrence of oral *H. pylori* infection. However, Ji et al. (2020) found no statistical differences in the detection of tongue coating *H. pylori* among infected, uninfected, and eradicated patients through high-throughput sequencing, which suggested that there is likely no substantial association between oral *H. pylori* and gastric *H. pylori*. In our study, we observed a greater incidence of tongue coating *H. pylori* among *H. pylori* positive patients compared to *H. pylori* negative subjects. Nevertheless, the observed disparity did not exhibit statistical significance (*p* > 0.05). It was worth mentioning that the presence of *H. pylori* on the tongue coating significantly exhibited a substantial decrease following *H. pylori* eradication (*p* < 0.01). The decrease in *H. pylori* presence may be explained by two factors. Firstly, the use of antibiotics to treat *H. pylori* in the gastric tract inadvertently leads to the elimination of *H. pylori* in the oral cavity as well (Bago et al., 2011). Secondly, it is possible that *H. pylori* in the oral cavity is either temporary or exists as nonviable bacterial fragments. The abundance or scarcity of *H. pylori* in the oral cavity is significantly impacted by its identified depth and specific location.

### 4.2. Effect of *Helicobacter pylori* infection on oral microbiota

The consistency of the results on the impact of *H. pylori* infection on oral bacteria diversity is not entirely uniform, perhaps due to regional disparities, variances in sample sources, and discrepancies in sample sizes. Multiple studies have demonstrated that there are no significant disparities in the alpha and beta diversity of the oral microbial community between *H. pylori* positive and negative individuals (Li et al., 2021; Wu et al., 2021). Other research suggested that there are noticeable variations in the beta diversity of the salivary microbiota between *H. pylori* infected and uninfected subjects (Ji et al., 2020). In their study, Chua et al. (2019) conducted an investigation wherein oral cavity samples were obtained from a total of 24 subjects during daylight hours. The researchers observed that *H. pylori* positive patients exhibited a notably lower Shannon index compared to *H. pylori* negative population (*p* = 0.03). Furthermore, the study revealed the presence of distinct oral microbial community structures between the groups infected with *H. pylori* and those who were not infected. Otherwise, a study conducted by Hu et al. (2022), demonstrated that *H. pylori* positive patients had higher richness and diversity and better evenness of oral microbiota than normal controls. Furthermore, a significant distinction in clustering patterns was observed between the two groups (*p* < 0.05). The results of our study indicate that there was a higher richness and diversity of tongue coating microbiota in *H. pylori* infected patients compared to healthy controls (*p* < 0.05 and *p* > 0.05, respectively). Additionally, there were significant differences in the bacterial composition (*p* < 0.05). In a comprehensive study conducted by Yamashita and Takeshita (2017), sequencing techniques were employed to examine the composition of the salivary microbial community. The results of this investigation revealed a positive correlation between an elevated abundance of the salivary microbiota and compromised oral health, which suggests a potential impairment of oral health due to *H. pylori* infection, potentially mediated by an augmentation in the diversity of oral flora.

The study revealed that the two groups under investigation exhibited a constant presence of the five primary phyla, namely Bacteroidetes, Firmicutes, Proteobacteria, Fusobacteria, and Actinomycetes, in a specific order. This finding is in accordance with previous research (Ji et al., 2020; Hu et al., 2022). There were notable differences in the oral microbial makeup between the two groups. The results of the LEfSe analysis revealed that there were discernible taxonomic differences between the two groups, which is consistent with previous research findings (Ji et al., 2020; Hu et al., 2022). However, these findings contradict the results reported by Li et al. (2021). In our study, we observed significant enrichment of *Fusobacterium* and *Haemophilus* in *H. pylori* infected individuals. *Fusobacterium* is classified as a commensal oral bacterium; however, *Fusobacterium nucleatum* is hypothesized to be linked to the development of periodontal disease (Signat et al., 2011). *Haemophilus* is generally regarded as non-pathogenic to humans; nonetheless, there have been documented cases linking *Haemophilus parainfluenzae* to endocarditis (Revest et al., 2016) and pseudomembranous colitis (Liu Q. et al., 2022). *Porphyromonas*, together with *Fusobacterium* and *Haemophilu*s, is recognized as a significant indicator of tongue coating microbiota in *H. pylori* infected individuals, as shown in our results. Ahn et al. (2012) have suggested that specific microorganisms found in the mouth cavity, such as *Porphyromonas* and *Fusobacteriaceae*, have the potential to disturb the microecology within the stomach and contribute to the development of chronic inflammation, which may serve as a potential precursor to gastric cancer. In a study conducted by Hu et al. (2015), notable reductions were observed in the relative prevalence of *Porphyromonas*, *Fusobacterium*, and *Haemophilus* among individuals diagnosed with stomach cancer. In contrast, Stasiewicz and Karpinski (2022) have established a notable correlation between *Porphyromonas gingivalis*, a widely recognized oral pathogen, and *Fusobacterium nucleatum*, with the occurrence of oral squamous cell carcinoma. There is a suggestion that infection with *H. pylori* could potentially influence the colonization of oral bacteria, hence potentially impacting the overall health of the oral cavity.

Through our analysis of the oral microbiota in *H. pylori* positive patients and healthy controls, we have identified variations in the expression of signaling pathways. *H. pylori* infection has the potential to influence the gene expression of tongue fur flora, which may establish a connection between *H. pylori* infection and the development of cardiovascular diseases and metabolic diseases such as lipid metabolism. Prior studies (Chen et al., 2020; Sun et al., 2023) have provided evidence of a correlation between *H. pylori* infection and metabolic conditions, including dyslipidemia, hypertension, diabetes, and cardiovascular diseases. Nevertheless, the precise mechanism remains uncertain and could potentially be associated with chronic inflammatory responses, endothelial damage, and hyperhomocysteinemia induced by *H. pylori* infection (Vijayvergiya, 2015). Potential areas of investigation in future research may involve examining the correlation between *H. pylori* infection and metabolic illnesses, focusing on a microecological perspective.

The precise mechanism underlying the impact of *H. pylori* infection on oral microbiota composition and metabolism has yet to be fully elucidated. Insufficient research has been conducted on this topic; however, it may be linked to modifications in intragastric acidity, virulence factors, cytokines, and the immunological response of the host. A previous study has shown that the use of PPI can have an impact on oral flora (Mishiro et al., 2018). It is hypothesized that *H. pylori* infection may lead to the production of significant quantities of urease, an enzyme responsible for the breakdown of urea into carbon dioxide, hence reducing intragastric acidity. On the other hand, the virulence factors associated with *H. pylori*, including CagA and VacA, have the ability to consistently trigger cellular inflammatory reactions, leading to a reduction in stomach acid output. The modification of gastric acid secretion has the potential to result in a reduction in oral PH, which can have implications for the composition of the oral microbial population. The impact of *H. pylori* infection on the intestinal flora is mediated by this particular mechanism, as observed in the study conducted by Iino and Shimoyama (2021). Furthermore, existing evidence suggests that the presence of *H. pylori* infection triggers the activation of cytokines inside the inflamed gastric mucosa. These cytokines, such as TNFα and IL-6, play a crucial role in the development of metabolic diseases (Buzas, 2014). Hence, we propose the presence of a link between *H. pylori* infection and alterations in the metabolic pathways of the oral microbiota. The association between *H. pylori* infection and changes in host immunity has been postulated (Cheok et al., 2022). Therefore, any modification in the immune response has the potential to destabilize the oral microbial ecology.

### 4.3. Effect of *Helicobacter pylori* eradication on oral microbiota

Following the successful *H. pylori* eradication, we observed a significant decrease in alpha diversity and a notable alteration in the composition of the oral microbiota. These differences did not fully revert to the baseline level at W6. Additionally, notable discrepancies were seen in the signaling pathways of the oral microbiota at W0, W2, and W6. In summary, *H. pylori* eradication with vonoprazan-amoxicillin regimen has been observed to lower the diversity of tongue coating flora and impact its structure and function. Despite undergoing therapy, the flora exhibited only partial recovery within a limited timeframe, failing to reach the levels observed before eradication. The use of bismuth-containing quadruple therapy, intended for *H. pylori* eradication, has been demonstrated to result in a reduction in alpha diversity and alterations in the composition of the salivary microbiota (Ji et al., 2020). Jakobsson et al. (2010) observed alterations in oral microbiota for a period of 4 years after *H. pylori* eradication with a 7-day triple therapy and found that the diversity decreased immediately after eradication, then gradually returned to the original level over time. *H. pylori* eradication regimen consists mainly of antibiotics and PPI. Previous studies have shown that the use of antibiotics (Abeles et al., 2015) and PPI (Mishiro et al., 2018) may have an impact on oral flora, aligning with the aforementioned discoveries. Alternatively, it seems that the magnitude of the impact is associated with the length of treatment time and amount of antibiotics and PPI administered. Hu et al. (2022) investigated the efficacy of low-dose amoxicillin (2.0 g daily) and high-dose amoxicillin (3.0 g daily) combined with vonoprazan for 7 or 10 days to eradicate *H. pylori*, then found the salivary flora diversity was not affected and the change in oral microbial community structure was minimal after eradication, which differs from our findings. The observed discrepancy could potentially be attributed to the prolonged length of therapy and the administration of a higher dosage of the medicine in our prescribed regimen.

There was a consistent drop in the relative abundance of Firmicutes and *Lactobacillus* following *H. pylori* treatment. The observed decline in the relative abundance of Firmicutes was found to be statistically significant, aligning with the findings reported by Hu et al. (2022). Periapical periodontitis is attributed to the presence of polymicrobial infections, characterized by the coexistence of many bacterial species. Among them, Firmicutes have been identified as the most prevalent and abundant (Korona-Glowniak et al., 2021). At the genus level, there was a notable drop in the relative abundance of *Lactobacillus*, a bacterium that has been associated with the progression of dental caries (Zhang and Xu, 2022). The eradication of *H. pylori* has the potential to impede the proliferation of oral infections.

A prior study (Pellicano et al., 2020) has found an association between *H. pylori* infection and the occurrence of type 2 diabetes mellitus, autoimmune diseases, neuropathy, and metabolism-related diseases. Moreover, some studies have reported the potential improvement of glycemic control in type 2 diabetes mellitus and symptom control in Parkinson’s disease with *H. pylori* eradication (Pierantozzi et al., 2006; Song et al., 2021). Nevertheless, there is still limited comprehension of the pathophysiology of these disorders, and the precise impact of *H. pylori* infection and its eradication remains uncertain. Further research is necessary to fully explore these relationships. The findings of our research suggest that the eradication of *H. pylori* can impact the expression of signaling pathways associated with the aforementioned diseases. This provides an opportunity to investigate the connection between *H. pylori* and metabolic disorders, immune disorders, and neurodegenerative conditions from a microecological standpoint.

### 4.4. Probiotics supplementation could not reduce the disturbance of oral flora induced by *Helicobacter pylori* eradication

The results of our study indicate that there were noticeable differences in the alpha diversity, compositional structure, and metabolic pathways of the tongue microbiome before and after the eradication of *H. pylori*. Furthermore, it can be observed that the level of variation was similar in both the groups receiving probiotics and receiving the placebo. This suggests that the simultaneous administration of probiotics did not provide any relief in terms of the disruption to the structure and function of the oral flora. Probiotics are bacteria that provide benefits to the host organism, and numerous studies have demonstrated the efficacy of co-administering probiotics in the eradication of *H. pylori* for the regulation of gastrointestinal flora (Kakiuchi et al., 2020; Yuan et al., 2021; He et al., 2022). Nevertheless, there remains a divergence of opinions regarding the precise function of probiotics in the context of oral microecology. The literature has demonstrated that probiotics possess the ability to disturb oral pathogenic biofilms and uphold a state of equilibrium within the oral microecology (Nguyen et al., 2021; Radaic et al., 2022). For instance, *Bifidobacteria* have the ability to impede the proliferation of *Porphyromonas gingivalis* (Jasberg et al., 2016). In a recent meta-analysis conducted by Liu J. et al. (2022), a total of 11 randomized controlled trials were examined to assess the effects of probiotics on oral flora. The findings of this analysis indicated that there was no significant difference observed in the levels of gingival inflammation and the presence of oral pathogens, such as *Porphyromonas gingivalis*, *Prevotella intermedia*, and *Fusobacterium nucleatum*, between the groups receiving probiotics and those receiving the placebo. Dassi et al. (2018) conducted a study to analyze the impact of probiotics on oral microbiota in healthy volunteers through 16S amplicon sequencing before, during, and after the administration of probiotics. The results showed an increase in the diversity of the salivary microbiota, but the overall structure remained unaltered. To far, there has been a scarcity of research investigating the impact of probiotics on oral flora during the *H. pylori* eradication. A randomized, placebo-controlled trial (He et al., 2022) investigated the impact of probiotics combined with bismuth quadruple therapy on the oral microbiota. The study revealed that there was no statistically significant disparity in the variety of salivary flora between the groups administered with probiotics and those given the placebo. However, it did notice a more restricted impact on the organization of the microbial community. The probiotics group showed a notable decrease in the prevalence of harmful microorganisms such as *Porphyromonas*, *Actinobacillus*, and *Prevotella*. Nevertheless, the group administered with probiotics in our study did not exhibit a reduced disruption in terms of diversity, structural composition, and function of the tongue flora, and we took into account the following factors: (1) The utilization of dual therapy in this study to eradicate *H. pylori*, which solely involved the use of one antibiotic, inherently resulted in a minor impact on the composition of oral flora (Hu et al., 2022). (2) The guidelines for the administration of *Bifidobacterium bifidum* tetrapartum tablets indicate that penicillin hampers the viability of live bacteria. Although, in our study, patients were instructed to take amoxicillin and the probiotics at least 2 h apart. (3) Probiotics gain entry into the gastrointestinal tract *via* the oral cavity, where the potential for colonization by probiotics may be limited. (4) The oral microbiota is significantly influenced by local oral hygiene practices, and this particular source of variability posed challenges in maintaining control during our clinical study.

## 5. Conclusion

In conclusion, the study determined that there was no substantial correlation between oral and stomach *H. pylori*. However, it was observed that eliminating gastric *H. pylori* seemed to enhance the elimination of oral *H. pylori*. *H. pylori* infection increased the richness of the oral microbiota and altered its composition. Following vonoprazan-amoxicillin eradication therapy, there was a notable reduction in the diversity of tongue flora and subsequent alterations in its composition, which could not be fully restored within a short period. The proliferation of oral pathogens (including Firmicutes and *Lactobacillus*) exhibited restricted growth subsequent to the *H. pylori* eradication. Additionally, *H. pylori* infection and eradication may have an impact on the metabolic pathways expressed in the tongue microbiota. These pathways are associated with various health conditions, such as cardiovascular disease, abnormal lipid metabolism, metabolic diseases, immune disorders, and neurodegenerative pathologies. However, the clinical importance of this phenomenon remains to be elucidated. The addition of probiotics did not result in a reduction of the disturbance to the oral microbiota produced by *H. pylori* eradication.

## 6. Limitation

Firstly, our study did not consider the impact of diet, geography, and oral health status on oral microbiota. Secondly, this study is single-centered and has a small sample size, which may result in disparities with other studies. Larger studies are warranted to clarify these discrepancies. Additionally, we did not monitor long-term tongue flora changes following *H. pylori* eradication. In addition, it is unclear what clinical significance the effects of *H. pylori* infection and eradication have on oral microbiota signaling pathways, as shown in this study. Therefore, future studies should utilize larger sample sizes and longer follow-up times to further investigate the interactions between *H. pylori* infection, *H. pylori* eradication, and the oral microbiota.

## Data availability statement

The datasets analyzed for this study can be found in the National Center for Biotechnology Information (NCBI) (https://www.ncbi.nlm.nih.gov/) with the accession number PRJNA1001062.

## Ethics statement

The studies involving humans were approved by the Medical Ethics Committee of the Nanjing First Hospital (KY20220314-05). The studies were conducted in accordance with the local legislation and institutional requirements. The participants provided their written informed consent to participate in this study.

## Author contributions

RP: Data curation, Formal analysis, Methodology, Writing – original draft. ZZ: Supervision, Writing – review & editing. YQ: Data curation, Writing – original draft. WC: Data curation, Writing – original draft.

## Funding

The author(s) declare that no financial support was received for the research, authorship, and/or publication of this article.

## Conflict of interest

The authors declare that the research was conducted in the absence of any commercial or financial relationships that could be construed as a potential conflict of interest.

## Publisher’s note

All claims expressed in this article are solely those of the authors and do not necessarily represent those of their affiliated organizations, or those of the publisher, the editors and the reviewers. Any product that may be evaluated in this article, or claim that may be made by its manufacturer, is not guaranteed or endorsed by the publisher.

## Figures and Tables

**Table:** 

*H. pylori*	*Helicobacter pylori*
BtT group	Vonoprazan-amoxicillin regimen plus probiotics
PT group	Vonoprazan-amoxicillin regimen plus the placebo
NT group	*H. pylori* negative group
W0	Before *H. pylori* eradication
W2	After *H. pylori* eradication
W6	At confirmation of *H. pylori* infection cure
Bid	Twice a day
Tid	Three times a day
Qid	Four times a day
16S rRNA	16S ribosomal RNA
PCR	Polymerase Chain Reaction
CTAB	Cetyltrimethylammonium Bromide
ASVs	Amplicon Sequence Variants
QIIME	Quantitative Insights Into Microbial Ecology
PCoA	Principal Coordinate Analysis
PLS-DA	Partial Least Squares Discriminant Analysis
LEfSe	Linear discriminant analysis effect size
PICRUST2	Phylogenetic investigation of communities by reconstruction of unobserved state
KEGG	Kyoto Encyclopedia of Genes and Genomes
LDA	Linear Discriminant Analysis
13C-UBT	13C urea breath test
PPI	Proton pump inhibitor
P-CAB	Potassium competitive acid blockers
pH	Potential of hydrogen

## Footnotes

^1^ https://www.kegg.jp/
