# Supplementary material for: The impact of Helicobacter pylori eradication with vonoprazan-amoxicillin dual therapy combined with probiotics on oral microbiota: a randomized double-blind placebo-controlled trial
Source: Front Microbiol. 2023 Oct 2;14:1273709. doi: 10.3389/fmicb.2023.1273709 (PMC10577438; doi:10.3389/fmicb.2023.1273709)
Supplement: Supplementary file 1 [file Data_Sheet_1.pdf]

Supplement Table 1 The demographic characteristics between *H. pylori* positive and negative subjects. There were no significant differences between the BtT group, PT group, and NT group in demographic characteristics including age, sex, BMI index, smoking, and alcohol. Hp group: *H. pylori* positive subjects. NT group: *H. pylori* negative subjects.

	BtT group (n=29)	PT group (n=31)	NT group (n=20)	*P* value
Age, mean±SD, years	39.90±11.26	38.61±10.83	36.10±10.74	0.493
Sex, male/female	12/17	10/21	7/13	0.757
BMI, mean ± SD, kg/m^2	23.66±2.78	24.89±6.16	22.12±3.08	0.103
Smoking	4 (13.8%)	2 (6.5%)	0 (0.0%)	0.186
Alcohol	6 (20.7%)	6 (19.4%)	0 (0.0%)	0.068

Hp group: *H. pylori* positive subjects. NT group: *H. pylori* negative subjects.

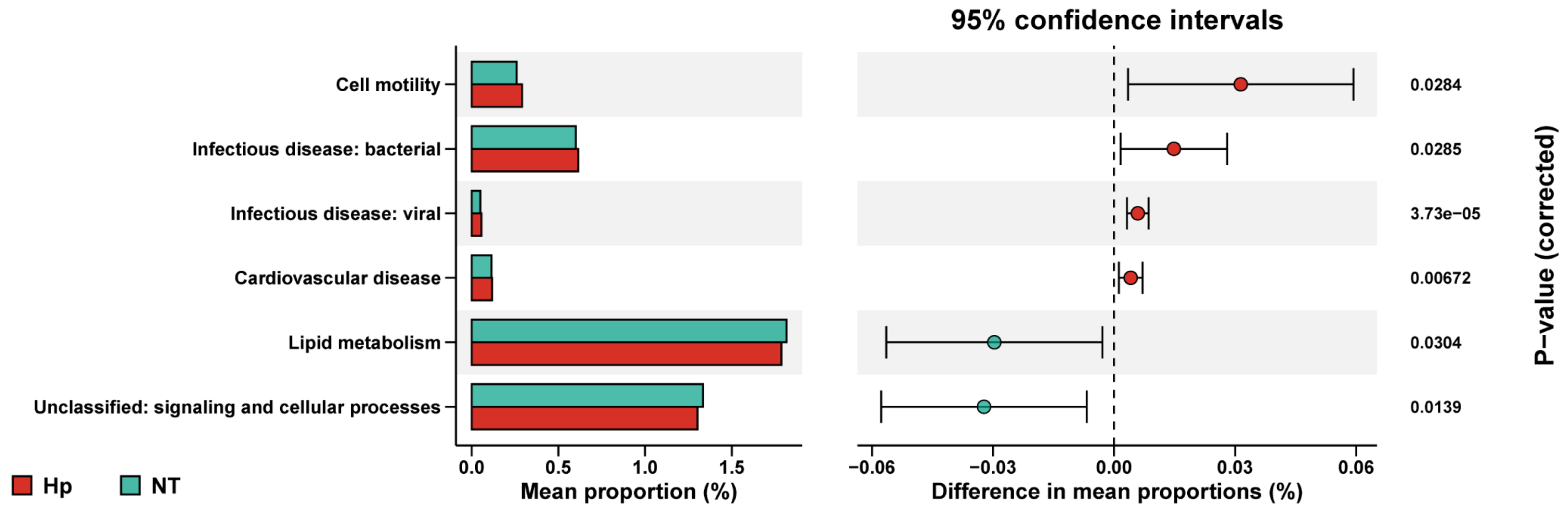

Supplement Figure 1 The KEGG analysis of the oral microbiota between H. pylori positive and negative subjects. Hp: H. pylori positive. NT: H. pylori negative

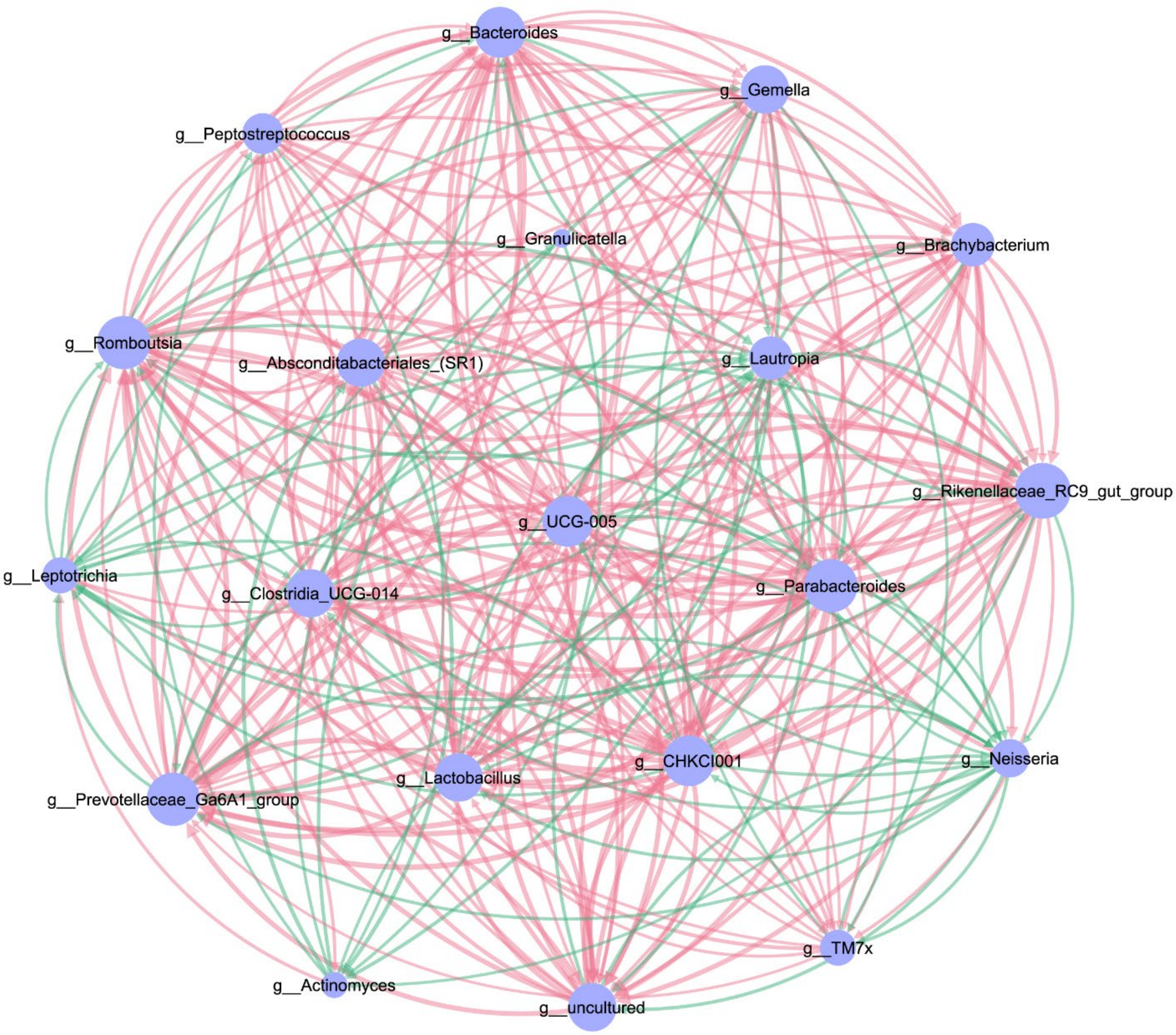

Supplement Figure 2 Co-occurrence network analysis of oral microbiota in the BtT group before and after H. pylori eradication. Each node represents a genus, and its size is proportional to its relative abundance. The edge color indicates the positive (red) and negative (blue) associations. The width of each line exhibits the strength of correlation among the genera. BtT group: vonoprazan- amoxicillin regimen plus probiotics; W0: before H. pylori eradication; W2: after H. pylori eradication.

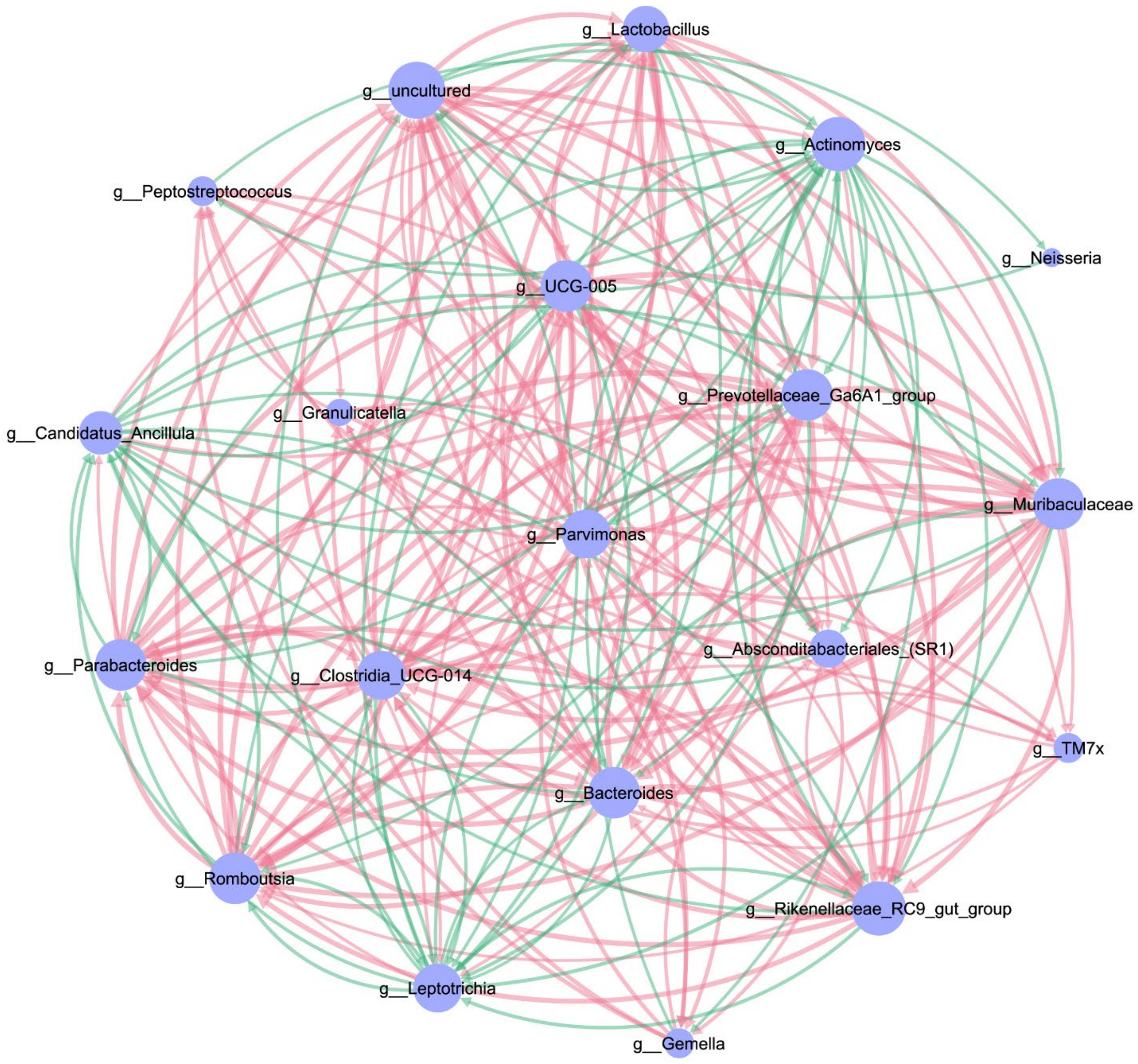

Supplement Figure 3 Co-occurrence network analysis of oral microbiota in the PT group before and after H. pylori eradication. Each node represents a genus, and its size is proportional to its relative abundance. The edge color indicates the positive (red) and negative (blue) associations. The width of 13C-UBT 13C urea breath test PPI proton pump inhibitor P-CAB potassium competitive acid blockers pH potential of hydrogen 20This is a provisional file, not the final typeset article each line exhibits the strength of correlation among the genera. PT group: vonoprazan-amoxicillin regimen plus the placebo; W0: before H. pylori eradication; W2: after H. pylori eradication.
